# Garlic Extracts: Effect of pH on Inhibition of *Helicobacter pylori*

**DOI:** 10.3390/life13071434

**Published:** 2023-06-24

**Authors:** Maria Teresa García, Jesus Manuel Garcia-Vargas, Luis Antonio Gómez Fernández, Pedro Cuevas, Ignacio Gracia

**Affiliations:** 1 Departamento Ingeniería Química, Faculty of Chemistry and Chemical Technologies, Universidad de Castilla-La Mancha, Avda. Camilo José Cela 10, 13004 Ciudad Real, Spain; teresa.garcia@uclm.es (M.T.G.); jesusmanuel.garcia@uclm.es (J.M.G.-V.); luisantonio.gomez@alvenpesalud.com (L.A.G.F.); 2 Histology Service, Hospital “Ramón y Cajal”, Planta 10 Izda., Ctra. Colmenar Viejo Km 9.1, 28034 Madrid, Spain; pedro.cuevas@hrc.es

**Keywords:** *Helicobacter pylori*, garlic extract, bacteriostatic, microbial growth, thiosulfinates, inhibition halo

## Abstract

The present work studies the influence of pH on the stability of thiosulfinates, compounds responsible for the bacteriostatic properties shown by ethanolic and acetonic garlic extracts (EGE and AGE) against the in vitro growth of *Helicobacter pylori* (Hp), a bacterium which is implicated in the etiology of diverse gastrointestinal diseases. The influence of pH and time on the stability of thiosulfinates and the microbiological activities of EGE and AGE has been evaluated at human body temperature (37 °C) and in a pH range of 0.9–4.7. A marked decrease in thiosulfinate concentration was observed in a relatively short time at pH values below 2.0. However, at pH values over 2.0, the samples maintained 70% of thiosulfinate concentration for 12 h. The inhibition halo diameters showed a maximum value at pH 2.50, with an inhibition halo of 28.94 ± 0.61 mm. The reduction in the activity at pH values below 2.0 was particularly remarkable. These results suggest that, for medical application, the pH of the selected extracts must only be maintained above 2 to maintain a high level of antibacterial activity. This fact would overcome the need for proton pump inhibitors and/or antibiotics during the treatment of Hp infections in human patients.

## 1. Introduction

Several cultures throughout the world have used garlic for medicinal purposes, with the earliest written evidence of this coming from ancient Egypt [1,2,3,4,5,6,7]. These healthy properties have been attributed to allicin, which was discovered in 1944 by Cavallito et al. [8]. Allicin is the most representative thiosulfinate (sulfur-based compounds present in many natural products like garlic or onions) and is extremely reactive. The healthy properties described for allicin include cardiovascular (blood pressure reducer, cholesterol reducer, blood viscosity reducer, vasodilator, antithrombotic), antioxidant, bactericide, and anti-tumor effects, among others [9]. However, the low stability of allicin [10], the absence of variety in industrial processes to produce natural garlic derivatives [11,12], and the lack of a connection between medical and chemical investigations regarding the properties of garlic have all contributed to ambiguous conclusions concerning garlic extracts. These factors have prevented the use of garlic products in the modern medical treatment of a wide range of diseases [13,14], despite the fact that their use as probiotics is recommended by some guidelines [15].

*Helicobacter pylori* (Hp) is the bacterium implicated in the etiology of a diverse range of gastrointestinal diseases, such as gastritis, gastric ulcers, MALT lymphoma and, in conjunction with other factors, even gastric cancer [16,17,18,19,20]. Several works have been published concerning the anti-Hp properties shown by aqueous garlic extracts [13,21,22,23]. Garlic extracts obtained with organic solvents have also shown anti-Hp properties [24], and the role of allyl-thiosulfinates in the inhibition of the in vitro growth of Hp has been demonstrated [25]. The mechanism described for the anti-Hp activity is based on the denaturalization of membrane proteins with -SH groups, which react with the thiosulfinate thiol group, preventing their proliferation via a bacteriostatic effect [25]. In addition, the effect of temperature and storage conditions on the stability of the principal allyl-thiosulfinate, allicin, has been established by thermodynamic equations [26]. However, before garlic extracts are used in live tests, their robustness must be determined in conditions that could affect their stability, e.g., the acidity of the stomach. These extracts could provide a natural alternative therapy to the current treatments that involve the use of a combination of antibiotics and cause problematic side-effects [27].

Gastric acid secretion is related to the duration and extent of Hp infections in humans [28,29,30]. Gastric pH during Hp infections can show long hyper- or hypochlorhydria states that persist for several months [31,32]. Table 1 summarizes data for the therapies used in modern treatments against Hp. Furthermore, over the short time spans, instantaneous variations of pH are produced by both changes of state in the colonization of Hp and normal digestive processes [32]. It is therefore important to establish the stability of the thiosulfinates in bioactive garlic extracts at different pH values that simulate gastric conditions and human body temperature.

The aim of this work is to determine the effect of gastric pH conditions at corporal temperature on allicin and other thiosulfinates in ethanolic and acetonic garlic extracts selected from a previous study [24], namely EGE and AGE. This investigation aims to ascertain whether thiosulfinates undergo decomposition induced by gastric pH conditions. Such information is required prior to performing live investigations, as well as to determine the bioactivity, formulation, and appropriate dosage of a natural remedy containing these extracts.

## 2. Materials and Methods

### 2.1. Materials

Ajoescar S.L. (Camino Hita, 0, 16660 Las Pedroñeras, Cuenca, Spain) supplied the purple garlic. The solvents employed were acetone (purity 99.5%) and ethanol (96% *v/v* purity). The remaining reagents included diallyl disulfide (purity 99%) as an internal standard, hydrochloric acid (purity 37%), and methanol (HPLC-isocratic-preparative) for instrumental analysis. All reagents were purchased from Panreac Química S.A. (Montplet & Esteban, S.A., Barcelona, Spain).

EGE and AGE were obtained in a stirred tank extractor under the conditions optimized in a previous study to maximize the yield of the process and the inhibition power against the in vitro growth of Hp [24]. The steps were as follows: For each extraction, 25 g of freshly milled garlic and 400 mL of the appropriate solvent (ethanol or acetone) were put into a 1 L extractor at 21 °C. The stirring system consisted of a jar-test Vittadini 6-P model Isco (Rome, Italy) with digital control of the stirring speed, which was set at 175 rpm. The extraction process was continued for 2 h, the mixture was filtered, and the solvent was evaporated using a Büchi R-114 rotary evaporator (Barcelona, Spain). HPLC analysis was used to determine the final thiosulfinate content using the procedure described below.

### 2.2. Characterization Techniques

#### 2.2.1. HPLC Analyses

The thiosulfinate content in the garlic was measured using a LC-10AD Shimadzu HPLC device (Duisburg, Germany), as described in [23]. A Supelcosil C18 column (150 × 4.6 mm, i.d. = 5 μm) supplied by Analisis Vínicos S.A. (Tomelloso, Spain) was used in combination with a methanol/water (50:50) mobile phase. An UV-vis detector tuned to 254 nm allowed us to detect the solute. The flow rate for the mobile phase was 1 mL/min, and the temperature was set at 25 °C; isocratic mode was used. As an internal standard, diallyl disulfide was utilized.

#### 2.2.2. Critical pH Determination

**Figure 1:**
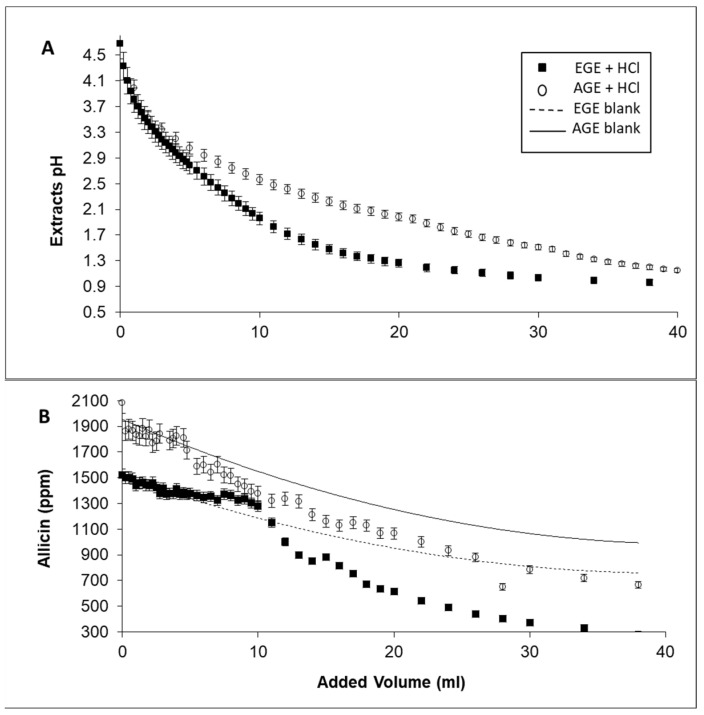
(**A**) Critical pH determination. Evolution of pH for EGE and AGE on addition of 1.03 N HCl. (**B**) Evolution of allicin concentration for EGE and AGE on addition of 1.03 N HCl (points) and deionized water (solid lines) in the blank experiment.

Critical pH is the pH level at which the protonation process results in a fast drop in allicin concentration. This was determined from the sudden change of slope (or the crossing of the two slopes) shown in Figure 1 for each thiosulfinate. This tendency variation is a result of the change from the normal dilution trend and the drastic change (drop in concentration) when an undesirable protonation reaction takes place.

The following method was used to assess how the concentration of thiosulfinate changed with fast pH variations: A 50 mL sample of solvent-free EGE or AGE was added to a 100 mL opaque glass flask, and the sample was vigorously agitated at 37 °C using an Agimatic-N magnetic-heater from P-Selecta (Abrera, Barcelona, Spain). A MicropH 2000 pH meter from Crison (Alella, Barcelona, Spain) was used to determine the pH of the garlic extract after adding various volumes of 1.03 N HCl, and the results were measured after 10 s. Samples (100 μL) of garlic extract were taken from the flasks for HPLC analysis for each volume added.

To determine the dilution effect, blank tests were performed by repeating the experiments with the addition of the same volumes of deionized water to the garlic extracts instead of HCl.

#### 2.2.3. Effect of pH on the Long-Term Stability of Allicin 

Thiosulfinate degradation was investigated through 12-h long time studies as follows: Six opaque glass flasks containing 50 mL of solvent-free EGE or AGE were put into a P-Selecta Unitronic S-320-200 thermostatic bath (Abrera, Barcelona, Spain) that had been set to 37 °C. By adding 5.71 N HCl, the pH of these extracts was kept constant within a range of 0.9 to 4. pH values were determined using a MicropH 2000 pH meter from Crison (Alella, Barcelona, Spain). The evolution of the thiosulfinate concentration was determined by taking samples (100 μL) of garlic extracts for HPLC analysis.

### 2.3. Microbiological Analysis

Extracts were prepared for microbiological analysis using a 10 mL glass vial filled with 2 mL of each sample mentioned in the previous paragraph and 1.5 mL of deionized water. This specific dilution was previously found to be ideal for achieving Hp growth inhibition zone widths (inhibition halos) of between 20 and 30 mm [24]). Vials were sealed and maintained at 6 °C in a Zanussi ZAC474 icebox (Albilux S.A., Barcelona, Spain) until microbiological tests were performed.

Hp isolates from consecutive patients undergoing upper digestive tract endoscopy were studied to determine their sensitivity to different garlic extracts according to the following microbiological process: A gastric antrum specimen was obtained endoscopically and sent to the laboratory for immediate processing. The specimen was streaked onto a Pylori-agar plate (bioMèrieux, Marcy l’Etoile, France) and then rolled onto a slide for Gram staining. The plate was incubated at 37 °C under microaerophilia (generated by a GENbox microaer, bioMèrieux, Marcy l’Etoile, France) for 7 days. The stained slide was examined and the presence of curved rod bacilli recorded. After 7 days of incubation, the plate was examined for growth. Bacterial colonies consistent with Hp grew as small, translucent colonies, which were confirmed as Hp by strong urease and oxidase reactions. The microorganism was then subjected to garlic extract sensitivity testing. A heavy inoculum was prepared by swabbing as much culture as possible and streaking it onto one Pylori-agar plate to test the sensitivity of the organism to garlic extracts. The Pylori-agar plates were refrigerated at 6 °C until the microbiological tests were performed. The microorganism was suspended in 2 mL of Brain Heart Infusion Broth to obtain a McFarland standard number of 5 (equivalent to 1.5 × 10^9^ bacterial suspension/mL). A swab was soaked in this suspension and then applied to the Pylori-Agar plates. Plates were left at 37 °C for 15 min. For each garlic extract, one 6-mm filter paper disc was loaded with 20 μL of the extract, allowed to dry at 37 °C in an incubator for 15 min, and then applied onto the plate previously swabbed with the microorganism. Plates were then incubated at 37 °C in a microaerophilia environment (Genbox generator envelopes, bio-Mérieux, France) for 7 days. After incubation, inhibition zones of Hp growth (also called inhibition halos) were observed, measured, and recorded [24].

For the storage experiment study, lyophilized samples were stored in sealed glass flasks and maintained at 4 °C in a commercial freezer for 2 to 2.5 years. After this period, the samples were rehydrated with deionized water and submitted to microbiological analysis according to the procedure previously described.

## 3. Results and Discussion

Individuals with Hp infections can suffer hyper- or hypochlorhydria over long periods [34,35]. For this reason, to determine the clinical potential of garlic-based bioformulations, it is crucial to evaluate the influence of pH and time on the stability of thiosulfinates. To determine the influence of pH, EGE and AGE solvent-free garlic extracts (Table 2) were subjected to variations of pH to simulate conventional digestive processes [36]. To determine the influence of time, kinetic tests at constant pH values, corresponding to stationary states during Hp colonization [37], were performed. Once the chemical influence of pH and time on thiosulfinate stability had been evaluated, the corresponding microbiological activity was determined.

Table 2 includes the initial pH values of the garlic extracts as well as a typical composition of the most representative thiosulfinates found in the extracts tested in the study.

### 3.1. Critical pH Determination

To determine the possible effect of simulated gastric pH on the chemical stability of the thiosulfinates, different volumes of 1.03 N HCl were added to samples of EGE an AGE according to the procedure described previously. The evolution of the pH of the garlic extracts with the continuous addition of acid is shown in Figure 1A. As observed, the evolution of pH was different for the two extracts, showing a more intense decrease for low quantities of added volume for the EGE curve. This was probably due to differences in the composition of the extracts.

**Figure 2:**
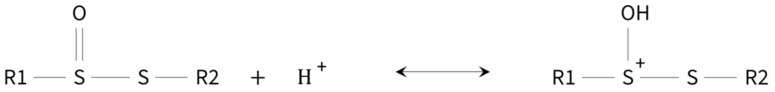
Protonation of thiosulfinates in acid media. (R1, R2 = Allyl, Methyl, Propenyl).

As noted previously, in acid media, the thiosulfinates can undergo protonation at the oxygen located on the sulfinyl sulfur atom, giving rise to an equilibrium that involves the generation of an electrophilic center and the partial destruction of the thiosulfinate [38], following the equilibrium represented in Figure 2. Therefore, a shift in the pH value of the media influences the concentration of thiosulfinates.

The concentrations present in EGE and AGE were analyzed by HPLC. Figure 1B represents the evolution of allicin concentration—the main and most active allyl-thiosulfinate in garlic extracts [25]—in EGE and AGE upon the addition of 1.03 N HCl (points) together with the blank experiments (solid lines). It can be observed that the allicin concentration for both curves is similar to that obtained in the blank experiments up to a certain point, called critical pH, where the allicin concentration decreases sharply after the addition of acid. This point indicates the pH from which the protonation reaction of sulfur compounds described in Figure 2 takes place because the concentration of protons in the reaction media favors the generation of products. These pH values are specific for each thiosulfinate and medium, a fact that explains the differences observed in Figure 1A. The same behavior was also observed for all thiosulfinates analyzed in EGE and AGE. Bearing in mind that the protonation reaction is undesirable because it decreases the concentration of bioactive thiosulfinates, it was necessary to quantify this effect.

**Figure 3:**
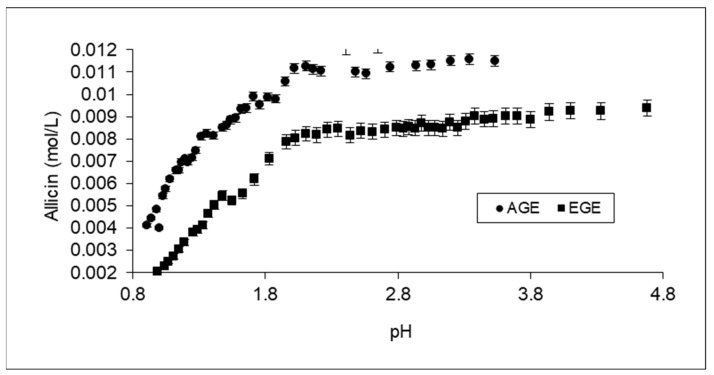
Evolution of the concentration of allicin against pH for EGE and AGE upon the addition of 1.03 N HCl.

The concentration of each thiosulfinate was plotted against the concentration of protons in the media, and the experimental data obtained for the thiosulfinates under investigation were fitted to linear regression. The pH range was divided into two zones: above and below the critical pH. The slopes of these plots below the critical pH show the amount of thiosulfinate that reacts per mol of protons (H+). As an example, Figure 3 shows the evolution of allicin concentration versus pH.

From this Figure, it can be seen that in the region with pH values higher than the critical pH, only a slight decrease in the thiosulfinate concentration occurred (right side of Figure 3). This change may have been due to a dilution effect on allicin concentration and a possible small conversion of thiosulfinate due to the reaction described in Figure 2. However, below the critical pH value, the concentration of allicin suddenly decreased rapidly due to a protonation reaction.

Table 3 shows the critical pH values obtained in the linear regression analysis of the evolution of the concentration of some of the different thiosulfinates present in the two garlic extracts studied (EGE and AGE) in the zones below the critical pH. The critical pH value and the coefficient value (r^2^) for the linear regression are shown for each extract.

It can be seen from the results in Table 3 that the thiosulfinates in EGE were more sensitive to the protonation reaction than those in AGE, as the slope values observed for the first ones were higher (in terms of absolute values), indicating a bigger shift in the concentration of thiosulfinates with pH. The one exception to this trend was allicin, which showed similar behavior in the two garlic extracts. It is worth noting that for all thiosulfinates analyzed, the critical pH values approximately corresponded to a pH value of around 2. This information is important for future medical applications, as it indicates that event instant variations of pH to values lower than 2 should be avoided. This aspect will be considered for further investigations in live tests, since the intragastric pH after a meal normally ranges from 1.5 to 4.5 in humans [36].

### 3.2. pH Effect on the Long-Term Stability of Allicin

**Figure 4:**
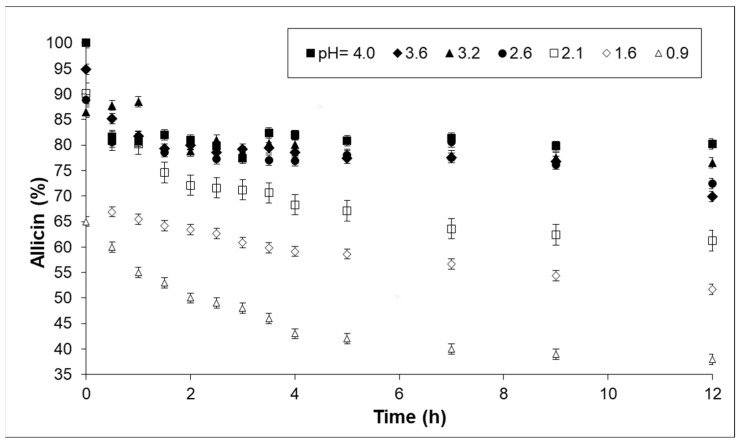
Evolution of allicin concentration as a function of time for AGE at various constant pH values.

To analyze the evolution of the concentration of thiosulfinates as a function of time at different constant pH, a set of experiments was performed at pH between 0.9 and 4 and at corporal temperature (37 °C) using EGE and AGE. These experiments were performed, according to the experimental procedure, over a period of 12 h in order to broadly simulate the time of human digestion. As a representative example of the thiosulfinates studied, the evolution of allicin concentration as a function of time for a sample of AGE at the different pH values studied is shown in Figure 4.

As expected, a decrease in allicin concentration was observed with time in all tests. This behavior was due to the occurrence of both protonation reactions in the acid media, as reported in Section 3.2, and thermal and oxidative degradation [26]. A marked decrease in thiosulfinate concentration was observed in a relatively short period of time at pH values below 2.0; this decrease occurred increasingly sharply with a further decrease in pH (blank dots in Figure 4). However, samples at pH values above 2.0 retained more than 70% of their original thiosulfinate concentration for 12 h. Analogous results were found for the other thiosulfinates analyzed in AGE and for the corresponding EGE extracts. The decrease of concentration observed for pH values lower than 2 clearly indicates that this limit should be considered when determining the formulation and dosage of a natural remedy using these selected extracts.

### 3.3. Microbiological Tests

To assess the anti-microbiological activity of extracts against the in vitro growth of Hp at different pH values, samples of EGE and AGE were analyzed according to the procedure described in Section 2.3.

**Figure 5:**
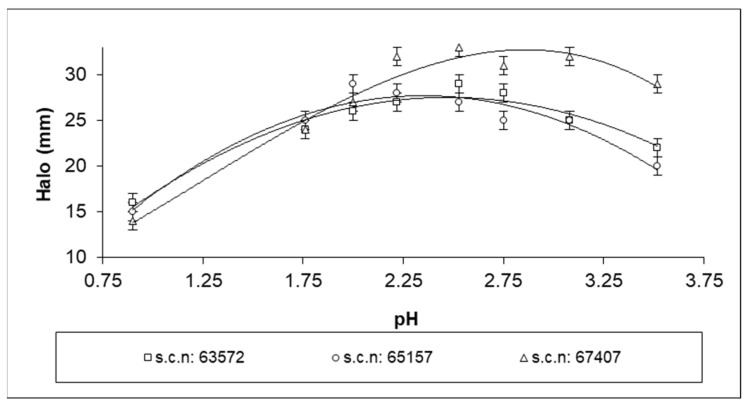
Diameters of the inhibition halos generated by AGE against pH for three different strains of Hp (s.c.n: strain clinical registration number).

Figure 5 shows the diameters of the inhibition halos produced by samples of AGE at the studied pH values for three different strains of Hp. The strain clinical registration numbers are indicated in the legend of this figure. The diameters of the inhibition halos showed maximum values at pH 2.3–2.8 (mean 2.5), i.e., 28.94 ± 0.61 mm S.D. This result may have been due to the higher stability of thiosulfinate concentration over pH 2.0, as reported in the previous section.

It is interesting to note that these results show that the best inhibition did not happen at the higher pH in conjunction with the maximal initial thiosulfinate concentration. This may have been because, in the total degradation of thiosulfinates, we must also consider thermal degradation [26], a process favored at higher pH values [37,38]. Thus, in the seven days over which the microbiological analyses were carried out, samples at higher pH values suffered more extensive thermal degradation. On the other hand, the degradation due to the protonation process, as represented in Figure 2, was not significant at pH values higher than 2.0, as described above. Similar results were obtained with EGE samples.

**Figure 6:**
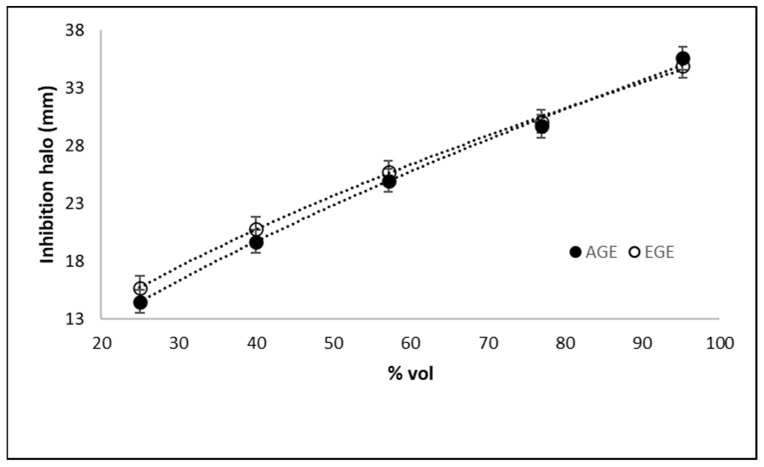
Effect of allicin concentration on the inhibition of the in vitro growth of *Helicobacter pylori* at pH 2.5.

Once the optimal pH for anti-Hp activity had been determined, a set of experiments was performed to determine the relationship between Allicin concentration and the inhibition halos for AGE and EGE at pH = 2.5 and 37 °C. To maintain the microbiological procedure, the different Allicin concentrations were obtained by dilution of the 20 μL original samples with volumes of mili-Q deionized water. Figure 6 shows a linear relationship in the range of concentrations tested for both extracts, indicating a dose-response effect. A regression of Figure 6 data led to Equations (1) and (2):

Halo AGE (mm)] = 1.6944 × C (% vol)^0.6646^ r^2^ = 0.9975 (1)

Halo EGE (mm)] = 2.3004 × C (% vol)^0.5966^ r^2^ = 0.9964 (2)

These equations will be useful in the formulation of an alternative natural therapy for the treatment of *Helicobacter pylori* infections and related diseases based on bioactive garlic derived products.

Considering that inhibition halos smaller than 10 mm are not significant according to microbiological criteria, from the data in Figure 6, the minimum inhibition concentration (MIC), corresponding to the minimal allicin concentration in the extracts required to produce inhibition activity, can also be determined. This MIC was a key parameter in the formulation of the dosage in the subsequent clinical testing of this alternative therapy. The MICs determined for AGE and EGE were:

MIC _AGE_= 14.46 (±1.73) % vol = 263 ppm allicin (3)

MIC _EGE_= 11.74 (±1.23) % vol = 178 ppm allicin (4)

**Figure 7:**
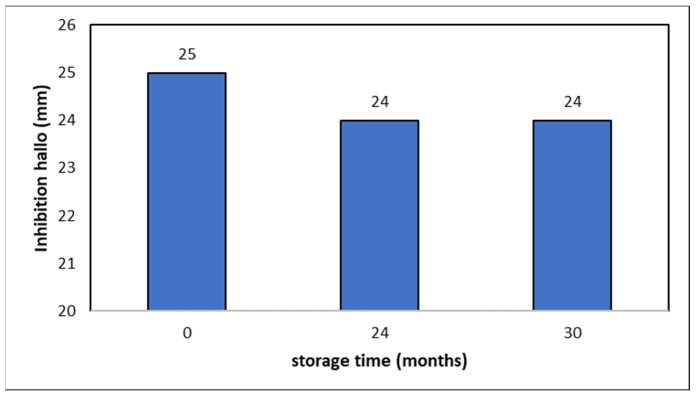
Storage stability of EGE samples. Inhibition Haloes after 2 years at pH 2.5 and 37 °C.

From a market-related perspective, a feasible industrial application must take into account other crucial factors, like storage stability. Since the minimum recommended expiry period in the EU for nutraceutical products is 2 years, AGE and EGE extracts were preserved by lyophilization under a patented process [9]. Lyophilized samples were stored in sealed glass flasks and maintained at 4 °C in a commercial freezer for 2 to 2.5 years. After this period, the sampled were rehydrated with deionized water and submitted to microbiological analysis according to the procedure described in Section 2.3. Figure 7 shows the inhibition halos for the fresh EGE samples and those corresponding to the preserved ones. As it can be observed, freeze-dried samples maintained practically the same inhibition activity to the original samples. The same results were obtained for the AGE samples. These facts suggest that our samples avoided one of the main problems related to the use of the natural matrixes in pharmacological industry, i.e., a lack of stability of activity over time, indicating them as potentially interesting candidates.

In order to assess the possible advantages of an alternative therapy, we considered the compounds used in the updated treatments against *Helicobacter pylori* (Table 1), in addition to AGE and EGE extracts, with respective inhibition halos of 27 and 30, obtained under the same experimental conditions. In the first column in Table 1 is a list of antibiotics, the most widely used generic PPI, omeprazole, and our extracts; the second column indicates the need to use a PPI; the third column shows the indications of each antibiotic, i.e., first or second line, triple, quadruple, or concomitant therapy. The table also notes the main side effects and, if determined, the inhibition haloes obtained under the same experimental conditions.

All antibiotics require the use of a PPI to maintain a pH of over 4 to ensure the efficiency of the treatment. From our previous results about critical and optimal pH, AGE and EGE extracts would not require the use of a PPI, given that their optimal efficiency is at normal stomach acidity conditions. This fact could prevent side effects in this specific treatment and would also reduce the omeprazole dosage for long-term therapy patients, thereby preventing serious diseases like osteoporosis [35]. In addition, an alternative therapy including natural AGE and EGE would avoid the use of traditional antibiotics, which have associated side effects. This alternative non-antibiotic therapy may also facilitate the reduction of antibiotic resistance for various bacteria and could be indicated for very persistent infections in patients with unsatisfactory treatment results or for those who are allergic to penicillin derivatives.

The in vitro inhibition halos of AGE and EGE were also compared in Table 1 to literature data obtained under the same experimental conditions as those applied in our work. In previous results, metronidazole did not show Hp inhibition, while Ciprofloxacin, a compound of the same family of Levofloxacin, showed high inhibition against Hp. This information suggests that our extracts are interesting candidates for further research as potential ingredients in a natural remedy against Hp infection.

## 4. Conclusions

Solvent-free garlic extracts obtained with ethanol and acetone inhibited the in vitro growth of Hp under simulated gastric pH conditions at human body temperature.

Variations of pH produced a protonation reaction involving the oxygen located on the sulfinyl-sulfur atom, decreasing the thiosulfinate concentration. This effect was particularly significant when the pH value passed below critical pH values. Microbiological tests showed a maximum inhibition at pH 2.5 and a sharp reduction in the activity at pH values below 2.0.

Our results were dose-dependent with allicin concentrations above the MIC values, and inhibition activity was maintained after two years of storage.

These results suggest potentially important medical applications of such extracts, avoiding the use of proton pump inhibitors during the treatment of Hp infections in human patients.

## Figures and Tables

**Table 1:** Compounds used in therapies against *Helicobacter pylori*.

Compound	PPI	Therapy [33,34]	Side Effects [35]	Halo
Clarithromycin	Yes	1 L, (3, C)	Nausea, vomiting, headache, and diarrhea	-
Amoxicillin	Yes	1 L, 2 L (3, C)	Diarrhea, nausea, and vomiting	-
Metronidazole	Yes	1 L, 2 L (3, 4)	Lack of coordination, blurred vision, pain, hot flushes, nausea, palpitations, depression, trouble speaking, and headache	0 [24]
Bismuth salts	Yes	1 L, 2 L (4)	Headache, nausea, pain, and diarrhea	-
Tetracycline	Yes	1 L, 2 L (4)	Sensitivity of skin to sunlight	-
Clarithromycin	Yes	2 L	Dizziness, headache	-
Levofloxacin	Yes	2 L	Diarrhea and insomnia	40 * [24]
Nitroimidazole	Yes	C	Headache, loss of appetite, nausea, diarrhea, heartburn, stomach cramping, constipation, metallic taste	-
Omeprazole		All, as PPI	Headache, vomiting, or diarrhea	-

PPI: requirement of a Proton Pump Inhibitor. Therapy: 1 L, 2 L first or second line; (3): triple therapy; (4): quadruple therapy; (C): concomitant therapy. (*) as Ciprofloxacin.

**Table 2:** Typical thiosulfinate analysis of EGE and AGE, as used in this study.

Concentration ^a^ (mol/L)	Al-SO-S-Al (allicin)	Me-SO-S-Me	Pr-SO-S-Me + Me-SO-S-Pr	Initial pH
EGE ^b^	0.0094	0.0462	0.0814	4.68
*AGE * ^b^	0.0129	0.0484	0.0885	4.00

^a^ Compound R1–SO–S–R2: R1, R2 = Al (allyl group), Me (methyl), Pr (propenyl). ^b^ EGE and AGE, ethanolic garlic extract and acetonic garlic extract; see experimental procedure. Average data from triplicate analysis corresponding to deviations under 5%.

**Table 3:** Critical pH determination for EGE and AGE extracts.

	EGE ^b^		AGE ^b^	
R1, R2 as R_1_–SO–S–R_2_^a^	Critical pH	r^2^	Critical pH	r^2^
Al-Al (Allicin)	1.87	0.966	1.92	0.989
Me-Me	2.07	0.988	1.29	0.991
Pr-Me + Me-Pr	2.13	0.971	1.59	0.995

^a^ Compound: R_1_, R_2_ = Al (allyl group), Me (methyl), Pr (propenyl). ^b^ EGE and AGE, ethanolic garlic extract and acetonic garlic extract; see experimental procedure.

## Data Availability

Supporting Data results are not included in archive data sets, but can be available by contacting authors.
